# Fatty Acid Profile, Mineral Composition and Heavy Metal Contamination in Brown Bear (*Ursus arctos*) Muscle: Implications for Human Health Risk Assessment

**DOI:** 10.1007/s12011-026-05273-5

**Published:** 2026-08-01

**Authors:** Peter Haščík, Matúš Rajský, Miroslava Kačániová, Adriana Pavelková

**Affiliations:** 1 Institute of Food Sciences, Faculty of Biotechnology and Food Sciences, Slovak University of Agriculture in Nitra, Tr. A. Hlinku 2, Nitra, 949 76 Slovak Republic; 2 Institute of Nutrition, Research Institute for Animal Production Nitra, National Agricultural and Food Centre, Hlohovecká 2, Lužianky, 951 41 Slovak Republic; 3 Institute of Horticulture, Faculty of Horticulture and Landscape Engineering, Slovak University of Agriculture in Nitra, Tr. A. Hlinku 2, Nitra, 949 76 Slovak Republic

**Keywords:** Trace elements, Muscle, Meat quality, FAs, Lipid indices, Risk assessment, *Ursus arctos*

## Abstract

This study evaluated the proximate composition, fatty acid profile, mineral composition, and heavy metal contamination of brown bear (*Ursus arctos*) muscle, with emphasis on its nutritional quality and potential health risks for consumers. Muscle samples (*musculus semimembranosus* and *musculus triceps brachii*; *n* = 10) obtained from free-ranging individuals in Slovakia were analysed using validated analytical methods. Brown bear muscle was characterised by high protein content (~ 22.5 g/100 g) and low intramuscular fat content (< 1 g/100 g), confirming its lean nutritional profile. The fatty acid composition was dominated by monounsaturated fatty acids, followed by saturated and polyunsaturated fatty acids. Lipid quality indices indicated favourable nutritional properties, including low atherogenic and thrombogenic indices and a PUFA/SFA ratio above recommended nutritional thresholds. However, the relatively high n-6/n-3 ratio reflected the omnivorous feeding ecology and seasonal physiological status of the analysed animals. Iron and zinc represented the predominant trace minerals, highlighting the nutritional value of the analysed muscle tissue. Most toxic elements (As, Cr, Cd, Hg) remained below analytical detection or quantification thresholds and were therefore interpreted descriptively. Lead (Pb) was detected at low mean concentrations; however, substantial inter-individual variability resulted in sporadically elevated values, most likely associated with secondary contamination from bullet fragments. Exposure assessment based on consumption of a 100 g portion and an upper-bound approach for left-censored data indicated negligible toxicological relevance for Cd and Hg, whereas Pb exposure increased considerably under maximum exposure scenarios. Overall, brown bear muscle represents a nutritionally valuable food source; however, continued monitoring of Pb contamination in game meat remains warranted.

## Introduction

Game-derived muscle tissue has attracted increasing attention as a nutritionally valuable and sustainable alternative to products originating from conventional livestock production systems. This perception is closely linked to the natural living conditions of wild animals, including unrestricted movement, diverse feeding behaviour, and the absence of intensive production practices such as growth promoters or prophylactic antibiotic use, all of which may influence muscle quality and chemical composition [1, 2]. Consequently, tissues from free-ranging animals are generally characterised by high protein content, low intramuscular fat levels, and favourable nutritional profiles; however, their composition remains inherently variable and reflects species-specific traits together with environmental and seasonal influences [3, 4]. In several European regions, including the Carpathian area, tissues from wild mammals have traditionally represented a supplementary food source associated with hunting practices and local consumption traditions. Although brown bear (*Ursus arctos*) muscle is not commonly commercialised, it may occasionally enter the human food chain through legally harvested animals or management-related removals, particularly within hunting communities.

From a compositional perspective, muscle tissue from wild species typically exhibits high moisture content and substantial proportions of high-quality proteins combined with relatively low-fat levels, contributing to lower energy value compared with conventional meats [3, 5]. Cholesterol concentrations are generally comparable to those reported in domesticated species, although considerable variability may occur depending on muscle type, age, nutritional status, and physiological condition of the animals [4]. These characteristics support the view of game-derived tissues as nutritionally advantageous components of the human diet, particularly with respect to dietary lipid composition and fat quality [5, 6].

The fatty acid profile represents one of the central determinants of the nutritional quality of muscle tissue. Compared with conventional livestock species, tissues from free-ranging animals often exhibit higher proportions of polyunsaturated fatty acids (PUFA) together with lower levels of saturated fatty acids (SFA) [7]. Particular attention has been devoted to the balance between n-6 and n-3 PUFA, as well as to lipid quality indices such as the atherogenic and thrombogenic indices, which provide integrated evaluation of the potential effects of dietary fats on cardiovascular health [8, 9]. In wild mammals, however, fatty acid composition is influenced not only by feeding ecology but also by seasonal physiological adaptations. In brown bears, pronounced metabolic changes associated with hyperphagia prior to hibernation and prolonged fasting during winter dormancy substantially affect lipid metabolism, energy storage, and fatty acid utilisation [10–12]. Consequently, seasonal and physiological variation may contribute considerably to differences in muscle lipid composition.

In addition to lipid characteristics, the mineral composition of muscle tissue contributes substantially to overall nutritional value. Tissues from wild animals are recognised as important sources of essential macro- and microelements, particularly iron, zinc, and copper, which occur in highly bioavailable forms [13–15]. At the same time, elemental composition is strongly influenced by environmental conditions, including soil chemistry, vegetation structure, trophic interactions, and anthropogenic activities, resulting in substantial spatial variability [16, 17]. This variability complicates direct comparisons among studies and highlights the importance of region-specific investigations.

From a toxicological perspective, the occurrence of potentially toxic elements in tissues from free-ranging wildlife remains an important issue. Elements such as cadmium (Cd), lead (Pb), and mercury (Hg) may enter the food chain through environmental contamination as well as hunting-related factors, including the use of lead-based ammunition [16, 18]. According to the European Food Safety Authority, chronic dietary exposure to these contaminants may pose health risks because of their cumulative toxic effects, while no safe exposure threshold has been established for Pb, particularly with respect to neurodevelopmental effects in children [19, 20]. Previous studies have demonstrated that lead fragments originating from ammunition may substantially contribute to contamination variability in game tissues intended for human consumption [21, 22]. Although skeletal muscle generally contains lower concentrations of heavy metals than metabolically active organs such as liver and kidney, as previously reported in cervids and other free-ranging wildlife species [22, 23], it represents the principal edible tissue and therefore requires direct evaluation.

Among wild mammals, the brown bear (*Ursus arctos*) represents a particularly interesting biological model because of its omnivorous feeding behaviour, high trophic position, extensive home range, and pronounced seasonal metabolic adaptations. In contrast to herbivorous game species, omnivorous mammals may exhibit greater heterogeneity in fatty acid composition, mineral profile, and contaminant burden due to more diverse dietary inputs and opportunistic feeding behaviour [2, 4]. Moreover, large carnivorous and omnivorous mammals are considered valuable bioindicators of environmental contamination because tissue concentrations of toxic elements may reflect both local geochemical conditions and anthropogenic influences within ecosystems [17, 24]. Despite the ecological and nutritional relevance of this species, available data regarding the composition of brown bear muscle tissue remain limited, particularly in Central European populations.

Therefore, the aim of the present study was to evaluate the proximate composition, fatty acid profile, lipid quality indices, mineral composition, and heavy metal burden of brown bear (*Ursus arctos*) muscle obtained from free-ranging individuals in Slovakia, together with a preliminary screening-level assessment of potential toxicological risk.

## Materials and Methods

### Experimental Design and Sample Collection

The biological material used in this study consisted of muscle samples obtained from ten free-ranging brown bears (*Ursus arctos*) originating from different hunting areas within the Slovak Republic. The animals inhabited natural environmental conditions without direct human management, and their diet reflected naturally available omnivorous food resources characteristic of the species and local habitat conditions.

The analysed individuals were removed from the population either through protective intervention carried out by the brown bear intervention team or under an exception from species protection granted by the Ministry of the Environment of the Slovak Republic to authorised hunting ground users. Sampling was conducted within the framework of scientific cooperation between Veľká Fatra National Park and the National Agricultural and Food Centre Lužianky. No animals were killed specifically for the purpose of this study.

Due to the protected status of the species and the opportunistic nature of sampling, the available dataset was necessarily limited and heterogeneous. The analysed group consisted of five males and five females originating from different localities across the Slovak Republic. The estimated age of the animals ranged from 2 to 13 years, reflecting the natural biological variability characteristic of free-ranging brown bear populations. Sampling was carried out during the autumn period prior to hibernation, when brown bears undergo intensive hyperphagic activity associated with seasonal energy accumulation. This physiological state may substantially influence muscle lipid composition and metabolic characteristics and should therefore be considered when interpreting the obtained results.

Following carcass processing, samples of thigh (*musculus semimembranosus*) and shoulder (*musculus triceps brachii*) muscles were collected approximately 24 h *post mortem*. The samples were trimmed of visible connective tissue and external contamination surrounding the wound channel where applicable, portioned, vacuum-packed, transported under refrigerated conditions, and subsequently stored at − 18 °C until analysis.

Because the present study was based on a relatively small number of opportunistically obtained individuals (*n* = 10), the results should be interpreted as preliminary data intended to provide an initial overview of the nutritional composition and contaminant burden of brown bear muscle in Slovakia rather than as representative values for the entire population. Nevertheless, considering the limited availability of biological material from large free-ranging carnivores, the dataset provides valuable baseline information for future ecological, nutritional, and toxicological investigations.

### Determination of Proximate Chemical Composition

The proximate composition of brown bear muscle samples, including moisture, crude protein, crude fat, and cholesterol content, was determined using an INFRATEC 1265 analyser (FOSS, Hillerød, Denmark). The instrument operates in transmittance mode within a wavelength range of 850–1050 nm. Prior to analysis, approximately 50 g of each sample was homogenised and placed into glass sample cups (90 × 90 × 15 mm). Each sample was scanned twice, and the spectra were expressed as log 1/T (T = transmittance), representing the average of five scanning positions. Results were expressed as g/100 g of fresh muscle, except for cholesterol, which was expressed as mg/100 g. All analyses were performed in triplicate.

The method was applied for comparative evaluation of proximate composition among the analysed muscle samples.

### Fatty Acid Determination

Total lipids were extracted from lyophilised samples using Soxhlet extraction with petroleum ether. The extraction procedure followed standard protocols, and fatty acid methyl esters (FAME) were prepared according to ISO 12966-2:2017. Fatty acid composition was expressed as a percentage of total identified fatty acids. The analysis was performed using a Tecator Soxtec System HT 1043 extraction unit (Gemini, Apeldoorn, The Netherlands). Analytical-grade reagents were used throughout the analysis, including petroleum ether (boiling range 40–65 °C), methanol, and n-hexane. A certified 42-component FAME standard mixture (Supelco 47885-U, Sigma–Aldrich, Germany) was used for identification and calibration.

### Lipid Quality Indices

Lipid quality indices were calculated to provide a nutritional interpretation of the fatty acid composition. The atherogenic index (AI), thrombogenic index (TI), hypocholesterolemic/hypercholesterolemic ratio (h/H), polyunsaturated/saturated fatty acid ratio (PUFA/SFA), and n-6/n-3 ratio were calculated according to the equations proposed by Ulbricht and Southgate [8]. The indices were calculated individually for each sample based on the relative proportions of fatty acids and subsequently expressed as mean ± standard deviation. These indices were used for comparative evaluation of lipid quality between muscle types.

### Determination of Trace Elements and Heavy Metals

Muscle samples intended for mineral and toxic element determination were homogenised prior to analysis. Approximately 5.5 g of tissue was subjected to wet mineralisation using a microwave digestion system (Ethos UP, Milestone Srl, Italy). Samples were digested in a mixture of 5 mL concentrated nitric acid (HNO₃) and 1 mL hydrogen peroxide (H₂O₂). The digestion programme consisted of gradual heating to 200 °C over 15 min, followed by a 15 min holding period and subsequent controlled cooling.

After digestion, the mineralised solutions were filtered and quantitatively transferred into volumetric flasks, then diluted to a final volume of 50 mL with deionised water. Concentrations of macroelements and microelements (Ca, P, Mg, Na, K, Fe, Zn, and Cu) together with selected toxic elements (As, Cr, Cd, and Pb) were determined using inductively coupled plasma optical emission spectrometry (ICP-OES). Instrument calibration was performed using a certified multi-element standard solution (Sigma-Aldrich, Switzerland).

Method accuracy and analytical reliability were verified using certified reference material (CRM–ERM^®^-CE278k, mussel tissue), and all measurements were performed in duplicate. The analytical procedure demonstrated satisfactory repeatability and acceptable recovery values for the analysed elements. Total mercury (Hg) concentration was determined separately using a direct mercury analyser (MA-3 Solo Mercury Analyzer, Nippon Instruments Corporation, Japan) without prior chemical digestion.

Analytical sensitivity thresholds applied during toxic element determination are presented in Table 1. For As, Cd, Cr and Pb, analytical reporting was based on the limit of detection (LOD), whereas Hg concentrations were evaluated using the limit of quantification (LOQ) because of the analytical characteristics of the direct mercury analyser.

**Table 1:** Analytical Sensitivity Thresholds Applied for Toxic Element Determination in Brown Bear (*Ursus arctos*) Muscle

Element	Reporting threshold (mg/kg ww)
Arsenic (As)	0.030
Cadmium (Cd)	0.0040
Chromium (Cr)	0.050
Lead (Pb)	0.010
Mercury (Hg)	0.0020

Values for As, Cd, Cr and Pb represent analytical limits of detection (LOD), whereas the value reported for Hg represents the limit of quantification (LOQ). ww = wet weight

### Statistical Analysis

All analytical determinations were performed in duplicate unless stated otherwise, and the obtained data are presented as mean ± standard deviation (SD). Prior to statistical evaluation, normality of data distribution was assessed using the Shapiro–Wilk test. Differences between the analysed muscle types (*musculus semimembranosus* and *musculus triceps brachii*) originating from the same individuals were evaluated using a paired Student’s *t*-test when assumptions of normality were satisfied. In cases where normal distribution was not confirmed, the non-parametric Wilcoxon signed-rank test was applied. Statistical significance was established at *P* < 0.05. Statistical analyses were performed using XLSTAT^®^ software (version 2018.5.52280, Addinsoft, New York, USA).

For toxic elements with concentrations below the analytical sensitivity thresholds summarised in Table 1, formal statistical comparison was not performed, and the results were interpreted descriptively. Because As, Cd, Cr, and Hg concentrations remained below analytical reporting thresholds in most samples, inferential statistical analysis was performed only for Pb concentrations.

For exposure assessment, an upper-bound approach was applied by replacing concentrations below the reporting threshold with the corresponding LOD or LOQ value, in accordance with EFSA recommendations for the evaluation of left-censored contaminant data.

Because the present study was based on a relatively limited number of opportunistically obtained free-ranging individuals originating from different localities and age categories, the statistical outcomes should be interpreted cautiously, particularly with respect to inter-individual biological variability. Furthermore, all analysed animals were sampled during the autumn pre-hibernation period, when seasonal metabolic adaptations characteristic of brown bears may additionally influence tissue composition and contaminant distribution.

## Results and Discussion

### Proximate Chemical Composition of Brown Bear Muscle

The proximate chemical composition of brown bear (*Ursus arctos*) muscle is summarised in Table 2. No statistically significant differences (*P* > 0.05) were observed between *musculus semimembranosus* and *musculus triceps brachii*, indicating relatively comparable distribution of the principal nutritional components between the analysed muscles. Moisture content ranged from 72.37 to 72.95 g/100 g, protein from 22.43 to 22.49 g/100 g, and intramuscular fat from 0.65 to 0.78 g/100 g, while cholesterol concentrations remained stable at approximately 45 mg/100 g.

**Table 2:** Proximate Chemical Composition of Brown Bear (*Ursus arctos*) Muscle (g/100 g)

Parameter	Thigh (musculus semimembranosus)	Shoulder (musculus triceps brachii)	*p*-value
Water	72.95 ± 1.90	72.37 ± 1.35	0.258
Total protein	22.43 ± 0.62	22.49 ± 0.61	0.720
Crude fat	0.78 ± 0.33	0.65 ± 0.50	0.297
Cholesterol (mg/100 g)	45.00 ± 5.10	45.00 ± 4.13	0.977

Values are presented as mean ± standard deviation (SD). Differences between muscle types were not statistically significant (*p* > 0.05)

The protein content observed in the present study (~ 22.5 g/100 g) corresponds well with values commonly reported for European game species. Previous investigations have shown that protein concentrations in wild mammals generally range between 20 and 25 g/100 g depending on species, muscle type, age, physiological condition, and environmental factors [3, 5]. Comparable protein levels have also been reported in wild boar and cervid species, supporting the nutritional relevance of brown bear muscle as a source of high-quality dietary protein [2, 3]. The absence of significant differences between the analysed muscles suggests that anatomical location exerted only a limited influence on the distribution of the protein fraction.

A notable characteristic of the analysed samples was the very low intramuscular fat content, which remained below 1 g/100 g in both muscles. Compared with available data for European game species, these values are situated at the lower end of the reported range. Wild boar muscle has previously been reported to contain approximately 0.7–2.8 g/100 g fat, whereas deer species generally exhibit intramuscular fat concentrations exceeding 1 g/100 g under comparable conditions [2, 3]. The relatively low lipid content observed in the present study may therefore reflect species-specific metabolic characteristics together with the physiological status of the analysed animals.

In free-ranging mammals, lipid deposition is strongly influenced by feeding regime, seasonal food availability, age, sex, and overall metabolic status [1, 2]. In brown bears, pronounced metabolic adaptations associated with autumn hyperphagia and preparation for hibernation substantially affect energy storage and lipid metabolism [10–12]. Because all analysed animals were sampled during the autumn pre-hibernation period, the obtained proximate composition likely reflects a specific physiological state rather than universally representative species characteristics. Additional biological variability may also be associated with the opportunistic nature of sampling and the relatively broad age range of the analysed individuals (2–13 years).

Moisture content remained within a relatively narrow interval (~ 72–73 g/100 g), which corresponds with values previously reported for wild game species, typically ranging between 70 and 76 g/100 g [3, 5]. This observation agrees with the well-established inverse relationship between water and lipid content in skeletal muscle tissue [19]. Cholesterol concentrations (~ 45 mg/100 g) were within the lower range reported for game species and comparable to values described for other wild mammals [4, 5]. Cholesterol content in skeletal muscle is known to vary depending on species, muscle type, nutritional condition, and metabolic status [4]. The relatively low concentrations observed in the present study therefore suggest that brown bear muscle does not represent a major dietary source of cholesterol compared with conventional red meats or some other game species.

The obtained results generally correspond with previous findings reported for brown bear muscle. Kelava Ugarković et al. [4] similarly described relatively high protein content together with low intramuscular fat concentrations, although compositional differences among studies may arise from variation in feeding ecology, geographical origin, season, and physiological condition of the analysed animals. Seasonal metabolic fluctuations are particularly important in large hibernating mammals because intensive pre-hibernation hyperphagia and subsequent fasting during hibernation may substantially alter nutrient metabolism and tissue composition [10, 11]. Consequently, direct comparisons among studies conducted during different seasons should be interpreted cautiously.

Overall, the analysed brown bear muscle was characterised by high protein content, low intramuscular fat levels, moderate moisture content, and relatively low cholesterol concentration. Compared with other European game species, the protein fraction appeared generally comparable, whereas the lipid fraction remained relatively low in the analysed samples. The absence of significant differences between the analysed muscles additionally suggests that proximate composition may be influenced more strongly by individual physiological and ecological factors than by anatomical location alone. Because the analysed animals were obtained opportunistically during the autumn pre-hibernation period, the findings should be interpreted as preliminary data reflecting a specific physiological context rather than definitive species-wide values.

### Fatty Acid Composition of Brown Bear Muscle

The fatty acid composition of brown bear (*Ursus arctos*) muscle is presented in Table 3. The analysed samples were characterised by a predominance of monounsaturated fatty acids (MUFA; ~45–46%) and saturated fatty acids (SFA; ~33–34%), whereas polyunsaturated fatty acids (PUFA) accounted for approximately 19% of total fatty acids. This distribution generally corresponds with lipid profiles previously reported in wild mammals, in which oleic (C18:1n-9), palmitic (C16:0), and stearic (C18:0) acids represent the dominant fatty acid fractions [1, 20]. Similar fatty acid patterns have also been described in brown bear muscle and other free-ranging omnivorous species [4].

**Table 3:** Fatty Acid Composition of Brown Bear (*Ursus arctos*) Muscle (% of Total Fatty Acids)

Fatty acid	Thigh (musculus semimembranosus)	Shoulder (musculus triceps brachii)	*p*-value
C12:0 (lauric)	0.10 ± 0.01	0.10 ± 0.01	0.694
C14:0 (myristic)	1.36 ± 0.05	1.38 ± 0.05	0.135
C16:0 (palmitic)	24.44 ± 0.23	24.33 ± 0.20	0.084
C17:0 (heptadecanoic)	0.31 ± 0.04ᵃ	0.27 ± 0.06ᵇ	0.003
C18:0 (stearic)	10.86 ± 0.28ᵃ	10.56 ± 0.26ᵇ	0.001
C18:1n-9 (oleic)	26.46 ± 6.20ᵃ	21.17 ± 9.37ᵇ	0.020
C18:1n-7 (vaccenic)	4.79 ± 0.16ᵇ	4.91 ± 0.17ᵃ	0.020
C18:2n-6 (linoleic)	12.82 ± 2.90	11.91 ± 2.27	0.264
CLA (conjugated linoleic acid)	0.14 ± 0.01ᵃ	0.13 ± 0.02ᵇ	0.005
C18:3n-3 (α-linolenic)	0.25 ± 0.03ᵃ	0.22 ± 0.05ᵇ	0.003
C20:1 (eicosenoic)	0.37 ± 0.10	0.32 ± 0.11	0.085
C20:4n-6 (arachidonic)	1.85 ± 0.26	1.75 ± 0.39	0.256
C20:5n-3 (EPA)	0.10 ± 0.02ᵃ	0.09 ± 0.02ᵇ	0.002
C22:5n-3 (DPA)	0.13 ± 0.01	0.13 ± 0.01	0.185
C22:6n-3 (DHA)	0.03 ± 0.01	0.03 ± 0.01	0.537
Σ SFA	34.41 ± 1.44ᵃ	33.35 ± 1.62ᵇ	0.005
Σ MUFA	46.00 ± 1.32	45.61 ± 1.41	0.172
Σ PUFA	19.14 ± 2.47	19.60 ± 1.44	0.354
n-3 PUFA	0.63 ± 0.09	0.63 ± 0.07	0.879
n-6 PUFA	16.17 ± 2.53ᵃ	14.75 ± 2.66ᵇ	0.046

Values are presented as mean ± standard deviation (SD). Different superscript letters (a, b) within a row indicate statistically significant differences between muscle types (*p* < 0.05)

Among individual fatty acids, oleic acid was the most abundant component, followed by palmitic and stearic acids, confirming the typical structure of intramuscular lipids observed in both wild and domesticated mammalian species [20]. The predominance of MUFA in the analysed samples is nutritionally relevant because monounsaturated fatty acids are generally associated with favourable effects on lipid metabolism and cardiovascular health [6–8]. Comparable fatty acid distributions were previously reported by Kelava Ugarković et al. [4], supporting the biological consistency of the obtained findings.

From a nutritional perspective, the relative proportions of SFA, MUFA, and PUFA represent important determinants of lipid quality. In the present study, the PUFA/SFA ratio (approximately 0.56–0.59) exceeded the minimum recommended value of 0.40 for balanced human nutrition [17]. In contrast, the n-6/n-3 ratio remained relatively high (approximately 23–26), indicating predominance of n-6 fatty acids. Similar values have been reported in omnivorous and monogastric species, whereas grazing ruminants typically exhibit lower n-6/n-3 ratios due to greater intake of α-linolenic acid-rich forage [21, 22]. Consequently, the fatty acid profile of brown bear muscle appears less favourable in this respect when compared with strictly herbivorous wild ruminants. According to Valsta et al. [6], nutritional evaluation of muscle lipids should consider not only total fat content but also the balance between individual fatty acid groups and their potential implications for cardiovascular health.

The fatty acid composition observed in the present study was probably influenced by the omnivorous feeding ecology and physiological status of the analysed animals. In contrast to ruminants, where ruminal biohydrogenation substantially modifies dietary fatty acids before tissue deposition, omnivorous species incorporate dietary lipids into tissues more directly [2]. Brown bears consume highly variable food resources, including plant material, fruits, invertebrates, vertebrate prey, and anthropogenic food sources, which may contribute to inter-individual variability in muscle lipid composition [2, 4]. Because all analysed individuals were sampled during the autumn pre-hibernation period, the obtained fatty acid profile likely reflects seasonal metabolic adaptations associated with hyperphagia and intensive energy accumulation prior to winter dormancy [10–12]. Previous studies have demonstrated that hibernating mammals undergo pronounced seasonal shifts in lipid metabolism and fatty acid utilisation, potentially affecting tissue lipid composition depending on physiological state and season [10–12].

Statistically significant differences (*P* < 0.05) between the analysed muscles were observed for several individual fatty acids, including C17:0, C18:0, C18:1n-9, C18:1n-7, CLA, C18:3n-3, and EPA. However, these differences did not substantially affect the overall distribution of the principal fatty acid groups (ΣSFA, ΣMUFA, ΣPUFA), which remained relatively comparable between *musculus semimembranosus* and *musculus triceps brachii*. This observation suggests that fatty acid composition was influenced more strongly by individual physiological and ecological factors than by anatomical location alone.

The relatively low concentrations of long-chain n-3 PUFA, particularly EPA and DHA, may indicate limited intake of n-3-rich dietary sources. Similar observations have been reported in omnivorous wildlife species and differ from fatty acid profiles typically observed in free-ranging herbivorous ruminants, which often contain higher concentrations of α-linolenic acid and its derivatives [21, 22]. Seasonal food availability may additionally contribute to variability in PUFA fractions because dietary composition in brown bears changes markedly throughout the year depending on habitat conditions and energetic demands [10, 24].

In addition to nutritional relevance, fatty acid composition may also influence oxidative stability and sensory properties of muscle tissue. Higher proportions of unsaturated fatty acids, particularly PUFA, increase susceptibility to lipid oxidation, potentially affecting flavour stability and shelf life [20]. At the same time, these fatty acids contribute to sensory characteristics commonly associated with game-derived muscle tissue [1, 6].

Overall, the obtained results indicate that the fatty acid composition of brown bear muscle generally corresponds with values previously reported for wild omnivorous mammals and reflects both feeding ecology and seasonal physiological adaptations. Although the PUFA/SFA ratio indicated acceptable lipid quality, the relatively high n-6/n-3 ratio should be considered when evaluating the nutritional significance of the analysed samples. Because the analysed animals were obtained opportunistically during the autumn pre-hibernation period, the obtained findings likely reflect a specific physiological and seasonal context rather than universally representative species characteristics.

### Lipid Quality Indices

Lipid quality indices of brown bear (*Ursus arctos*) muscle are presented in Table 4. No statistically significant differences (*P* > 0.05) were observed between *musculus semimembranosus* and *musculus triceps brachii* for the atherogenic index (AI), thrombogenic index (TI), hypocholesterolemic/hypercholesterolemic ratio (h/H), or PUFA/SFA ratio, indicating relatively comparable nutritional quality of the lipid fraction between the analysed muscles. A significant difference was detected only for the n-6/n-3 ratio (*P* < 0.05), although the magnitude of this difference remained limited.

**Table 4:** Lipid Quality Indices of Brown Bear (*Ursus arctos*) Muscle

Index	Thigh (musculus semimembranosus)	Shoulder (musculus triceps brachii)	*p*-value
AI	0.47 ± 0.05	0.46 ± 0.05	0.218
TI	1.06 ± 0.11	1.04 ± 0.10	0.196
h/H	1.94 ± 0.19	1.97 ± 0.20	0.284
PUFA/SFA	0.55 ± 0.07	0.57 ± 0.08	0.233
n-6/n-3	24.5 ± 3.8^a^	23.2 ± 3.5^b^	0.047

Values are presented as mean ± standard deviation (SD). Different superscript letters (a, b) within a row indicate statistically significant differences between muscle types (*p* < 0.05)

The obtained AI values (0.46–0.47) and TI values (1.04–1.06) reflected a favourable balance between saturated and unsaturated fatty acids. These indices are primarily influenced by the relative proportions of hypercholesterolemic saturated fatty acids, particularly lauric (C12:0), myristic (C14:0), and palmitic acids (C16:0), in relation to monounsaturated and polyunsaturated fatty acids [8]. In the present study, the relatively low contribution of these saturated fatty acids together with the predominance of MUFA contributed to the observed lipid quality indices. Comparable AI and TI values have previously been reported for brown bear muscle and other free-ranging game species characterised by relatively low intramuscular fat content and higher proportions of unsaturated fatty acids [4, 7]. According to Valsta et al. [6], nutritional evaluation of muscle lipids should consider not only total fat content but also the balance between individual fatty acid groups and their potential implications for cardiovascular health.

The PUFA/SFA ratio (0.55–0.57) exceeded the recommended threshold value of 0.40 proposed for balanced human nutrition [18], indicating acceptable fatty acid composition from a nutritional perspective. In contrast, the n-6/n-3 ratio remained relatively high (23.2–24.5), reflecting predominance of n-6 fatty acids in the analysed samples. Similar values have been reported in omnivorous and monogastric wildlife species, whereas lower n-6/n-3 ratios are typically observed in free-ranging herbivorous ruminants due to greater dietary intake of α-linolenic acid-rich forage [21, 22]. Consequently, although the analysed muscle samples exhibited generally acceptable lipid quality indices, the elevated n-6/n-3 ratio should be considered when evaluating their nutritional significance.

The calculated h/H ratio (1.94–1.97) indicated predominance of hypocholesterolemic fatty acids over hypercholesterolemic fatty acids. This ratio complements AI and TI by providing broader evaluation of the nutritional characteristics of the lipid fraction [8]. Comparable h/H values have also been reported in wild mammals with relatively low intramuscular fat content and higher proportions of unsaturated fatty acids [4, 6]. Interpretation of these indices in free-ranging wildlife species should nevertheless consider biological variability associated with feeding ecology, habitat conditions, physiological status, and seasonal metabolic adaptations.

The lipid quality indices observed in the present study were probably influenced by the omnivorous feeding behaviour and pre-hibernation physiological status of the analysed animals. In brown bears, autumn hyperphagia is associated with intensive energy accumulation and pronounced alterations in lipid metabolism prior to winter dormancy [10–12]. These physiological adaptations may affect both fatty acid distribution and the calculated lipid quality indices. Furthermore, because the analysed individuals originated from different localities and represented a relatively broad age range (2–13 years), additional inter-individual variability related to environmental conditions, dietary composition, and metabolic status may also be expected.

Compared with other game species, the lipid quality indices of brown bear muscle generally fall within the range reported for wild mammals [1, 4]. However, interspecies differences remain evident due to variations in digestive physiology and feeding ecology. In ruminants, ruminal biohydrogenation modifies dietary fatty acids before tissue deposition, whereas in omnivorous species dietary lipids are incorporated more directly into muscle tissue [2]. Consequently, omnivorous mammals such as brown bears may exhibit greater variability in fatty acid composition and lipid quality indices depending on habitat conditions and food availability.

From a nutritional perspective, the obtained lipid quality indices generally corresponded with current dietary recommendations. Because the analysed animals were obtained opportunistically during the autumn pre-hibernation period, the calculated indices likely reflect a specific physiological and seasonal context rather than universally representative species characteristics.

### Mineral Composition

The mineral composition of brown bear (*Ursus arctos*) muscle is presented in Table 5. Potassium was the predominant macroelement in both analysed muscles (312.7–333.9 mg/100 g), followed by sodium (57.6–65.3 mg/100 g) and magnesium (18.7–20.0 mg/100 g), whereas calcium and phosphorus were present at lower concentrations. Among the analysed microelements, iron (4.73–5.45 mg/100 g) and zinc (4.96–5.70 mg/100 g) represented the most abundant trace elements, while copper occurred at comparatively lower concentrations. Statistically significant differences (*P* ≤ 0.05) between *musculus semimembranosus* and *musculus triceps brachii* were observed only for Na, K, and Cu concentrations, with higher values detected in *musculus triceps brachii*.

**Table 5:** Mineral Composition of Brown Bear (*Ursus arctos*) Muscle (mg/100 g Wet Weight)

Element	Thigh (musculus semimembranosus)	Shoulder (musculus triceps brachii)	*p*-value
Ca	7.6 ± 2.01	8.3 ± 0.01	0.462
P	14.2 ± 3.00	13.1 ± 0.01	0.314
Mg	18.7 ± 2.00	20.0 ± 0.00	0.133
Na	57.6 ± 9.0ᵇ	65.3 ± 10.0ᵃ	0.047
K	312.7 ± 28.0ᵇ	333.9 ± 20.0ᵃ	0.049
Fe	5.45 ± 0.99	4.73 ± 1.09	0.139
Zn	5.70 ± 1.62	4.96 ± 1.08	0.248
Cu	0.09 ± 0.05ᵇ	0.27 ± 0.14ᵃ	0.001

Values are presented as mean ± standard deviation (SD). Different superscript letters (a, b) within a row indicate statistically significant differences between muscle types (*p* < 0.05)

From a nutritional perspective, the relatively high concentrations of iron and zinc represent one of the most important findings of the present study. The obtained values correspond well with concentration ranges previously reported for European game species, where iron concentrations commonly range from approximately 1.9 to 8.2 mg/100 g and zinc concentrations from 2.3 to 11.7 mg/100 g depending on species, habitat conditions, and physiological status [5, 24]. Comparable mineral profiles have also been reported in wild boar and cervid species from Central Europe [14, 15], confirming that muscle tissue from free-ranging mammals may represent an important dietary source of highly bioavailable trace elements. The nutritional relevance of these findings is additionally supported by the high biological availability of heme iron in muscle tissue compared with plant-derived iron sources [13].

The mineral composition observed in the analysed samples was probably influenced by the omnivorous feeding ecology and environmental exposure characteristic of brown bears. Unlike herbivorous game species, omnivorous mammals obtain minerals from a broader spectrum of dietary sources, including plant material, invertebrates, vertebrate prey, and anthropogenic food resources [2, 4]. Consequently, mineral concentrations in brown bear muscle may reflect not only physiological regulation but also local environmental conditions, trophic interactions, habitat characteristics, and seasonal dietary variability [16, 17]. Because all analysed animals were sampled during the autumn pre-hibernation period, seasonal metabolic adaptations associated with hyperphagia and intensive nutrient accumulation may additionally have contributed to the observed mineral profile [10–12].

The predominance of potassium in the analysed muscles corresponds with the physiological distribution of macroelements in skeletal muscle tissue, where potassium functions as the principal intracellular cation. Similar distributions of major elements have consistently been described in wild mammalian species regardless of feeding ecology [1, 24]. In contrast, the relatively lower phosphorus concentrations observed in the present study compared with some other game species may reflect differences in muscle metabolism, dietary composition, or physiological status of the analysed animals [24].

Mineral composition in free-ranging wildlife is strongly associated with environmental conditions. Soil composition, vegetation, water availability, anthropogenic pollution, and regional geochemical characteristics all contribute to variability in elemental concentrations within animal tissues [16, 17, 25]. Large omnivorous and carnivorous mammals are additionally recognised as valuable bioindicators of environmental contamination because tissue mineral and trace element concentrations may reflect long-term ecological exposure within ecosystems [16, 26]. Previous studies performed in wildlife species from Central Europe have similarly demonstrated considerable inter-individual and regional variability in mineral composition associated with habitat conditions and feeding ecology [16, 23, 25].

The relatively low copper concentrations observed in the present study are consistent with previous reports in wild mammals, where copper concentrations in skeletal muscle generally remain lower than those of iron and zinc because copper is preferentially accumulated in metabolically active organs such as the liver [25, 27]. Similarly, the absence of pronounced differences between the analysed muscles suggests that overall mineral distribution was influenced more strongly by individual physiological and environmental factors than by anatomical location alone.

Overall, the obtained results indicate that brown bear muscle represents a nutritionally valuable source of essential minerals, particularly iron and zinc, while simultaneously reflecting the natural variability characteristic of free-ranging omnivorous mammals. Because the analysed animals were obtained opportunistically from different localities during the autumn pre-hibernation period, the obtained values likely reflect a specific ecological and physiological context rather than universally representative species characteristics.

### Heavy Metal Concentrations and Health Risk Assessment

The concentrations of selected toxic elements in brown bear (*Ursus arctos*) muscle were generally low. Arsenic (As), cadmium (Cd) and chromium (Cr) remained below the analytical limit of detection (LOD) in all analysed samples, whereas mercury (Hg) concentrations were below the limit of quantification (LOQ). Lead (Pb) was the only toxic element quantified, with mean concentrations of 0.02 ± 0.05 mg/kg ww in *musculus semimembranosus* and 0.01 ± 0.03 mg/kg ww in *musculus triceps brachii*, without significant differences between muscles (*P* = 0.215). These findings suggest that skeletal muscle did not represent a major site of toxic element accumulation under the evaluated conditions.

This distribution pattern corresponds with observations previously reported in free-ranging wildlife species, where edible muscle tissue generally contains substantially lower concentrations of toxic elements than metabolically active organs such as the liver and kidneys [26–28]. In wildlife species, tissue distribution of toxic elements is influenced by toxicokinetic processes, metabolic activity, feeding ecology, age, physiological status, and environmental exposure [16, 25, 26]. Comparable findings have also been described in brown bears from the Western Carpathians, where elevated heavy metal concentrations were associated predominantly with internal organs rather than skeletal muscle tissue [29]. The absence of detectable As, Cd and Cr, together with Hg concentrations below the LOQ, indicates limited accumulation of these toxic elements in edible muscle tissue. However, because the analysed individuals originated from different localities across Slovakia and represented a broad age range (2–13 years), the obtained data should not be interpreted as universally representative for the entire brown bear population.

Seasonal physiological status may additionally influence contaminant distribution in large hibernating mammals. All analysed animals were sampled during the autumn pre-hibernation period, when brown bears undergo intensive hyperphagia associated with energy accumulation and altered metabolism prior to winter dormancy [10–12]. These physiological adaptations may affect tissue composition and contribute to inter-individual differences in contaminant concentrations.

Lead represented the most toxicologically relevant element detected in the analysed muscle samples. Although average Pb concentrations remained below the maximum level established by current European legislation for meat intended for human consumption (Regulation (EU) 2023/915) [30], marked heterogeneity among individual samples was observed. Importantly, the maximum detected Pb concentration reached 1.7010 mg/kg wet weight, markedly exceeding the current EU maximum limit of 0.10 mg/kg. This finding is toxicologically important because Pb is recognised as a non-threshold neurotoxic contaminant, particularly with respect to neurological development in children [19]. Consequently, even sporadic occurrence of highly contaminated samples may substantially contribute to dietary Pb exposure.

The elevated Pb concentrations observed in several individual samples most likely originated from secondary contamination associated with bullet fragmentation during hunting procedures. Similar contamination pathways have been widely documented in wild game species, where microscopic lead fragments originating from ammunition may remain distributed within tissues surrounding the wound channel and substantially increase measured Pb concentrations [18, 21, 22]. Although visibly damaged tissue surrounding the wound channel was removed prior to analysis, microscopic fragments may still persist within adjacent muscle tissue. The heterogeneous distribution of Pb among samples therefore likely reflects localised contamination associated with ammunition residues rather than uniform environmental exposure.

The dietary exposure assessment based on consumption of a 100 g portion of brown bear muscle provides additional context for toxicological interpretation (Table 6). For Cd, the estimated intake represented only a minor fraction of the tolerable weekly intake established by the European Food Safety Authority [20], indicating negligible toxicological relevance under the evaluated consumption scenario. Similarly, Hg concentrations remained below the LOQ in all analysed samples.

**Table 6:** Estimated Dietary Exposure to Cadmium (Cd) and Lead (Pb) Following Consumption of a 100 g Portion of Brown Bear (*Ursus arctos*) Muscle

Element	Muscle/scenario	Concentration (mg/kg ww)	Intake per 100 g portion (µg)	Adult exposure (70 kg bw) (µg/kg bw)	Child exposure (20 kg bw) (µg/kg bw)
Cd	Upper-bound scenario	0.0079	0.79	0.011	0.040
Cd	Maximum detected value	0.0180	1.80	0.026	0.090
Pb	Thigh mean	0.0200	2.00	0.029	0.100
Pb	Shoulder mean	0.0100	1.00	0.014	0.050
Pb	Maximum detected value	1.7010	170.10	2.430	8.505

Values are based on a screening-level exposure scenario assuming consumption of a 100 g portion of muscle tissue. Exposure is expressed both as absolute intake per portion and as body weight-adjusted exposure for an adult (70 kg bw) and a child (20 kg bw). For concentrations below the limit of detection (< LOD), an upper-bound approach was applied using the corresponding LOD values. ww—wet weight; bw—body weight

For Pb, mean exposure estimates remained relatively low, ranging from 0.014 to 0.029 µg/kg body weight in adults and from 0.050 to 0.100 µg/kg body weight in children. Under the maximum exposure scenario, however, estimated Pb intake increased substantially, reaching 2.430 µg/kg body weight in adults and 8.505 µg/kg body weight in children. Although average exposure levels remained low, the occurrence of highly contaminated individual samples may considerably increase dietary Pb exposure in specific cases. This observation is particularly relevant because no safe exposure threshold for Pb has been established, especially for sensitive population groups such as children and pregnant women [19]. Similar conclusions have been reported in risk assessment studies of game meat consumption, where sporadic intake of contaminated portions may substantially contribute to total dietary exposure [31].

From an ecological perspective, large omnivorous and carnivorous mammals are widely recognised as useful bioindicators of environmental contamination because tissue burdens of toxic elements may reflect both geochemical background and anthropogenic pressure within ecosystems [6, 16, 25]. Previous studies in wildlife populations from Central Europe have similarly demonstrated regional differences in heavy metal accumulation associated with habitat characteristics and environmental exposure [25, 26]. In brown bears from the Carpathian region, tissue contaminant concentrations have likewise been associated with local environmental conditions [28]. In this context, the relatively low average concentrations of Cd and Hg observed in the present study may indicate limited environmental contamination pressure in the analysed localities, at least with respect to skeletal muscle tissue. Nevertheless, the pronounced Pb heterogeneity additionally highlights hunting-related contamination as a major confounding factor in toxicological evaluation of game-derived muscle tissue.

Several limitations of the present study should be acknowledged. The analysed dataset was based on a limited number of opportunistically obtained free-ranging individuals originating from different localities and age categories. In addition, all animals were sampled during the autumn pre-hibernation period, which may have influenced both tissue composition and contaminant distribution due to seasonal metabolic adaptations characteristic of brown bears. Furthermore, potential secondary contamination associated with bullet fragmentation cannot be completely excluded despite removal of visibly damaged tissue surrounding the wound channel. Consequently, the obtained findings should be regarded as preliminary data providing an initial overview of the nutritional and toxicological characteristics of brown bear muscle in Slovakia.

Overall, the present results indicate relatively low average concentrations of most analysed toxic elements in brown bear muscle from Slovakia. However, the marked variability observed for Pb demonstrates that occasional occurrence of highly contaminated samples cannot be excluded. Consequently, the analysed tissue cannot be uniformly regarded as consistently low-risk material for human consumption. Practical measures such as careful removal of tissue surrounding the wound channel and replacement of lead-based ammunition with non-lead alternatives may substantially reduce dietary Pb exposure. Further investigations involving larger datasets, seasonal comparisons, and detailed evaluation of contamination pathways would help clarify the toxicological characteristics of brown bear muscle under different ecological conditions.

## Conclusion

Brown bear (*Ursus arctos*) muscle was characterised by high protein content, low intramuscular fat levels, favourable lipid quality indices, and substantial concentrations of essential minerals, particularly iron and zinc. Most analysed toxic elements (As, Cr, Cd, Hg) remained below analytical detection or quantification thresholds, indicating limited accumulation in skeletal muscle tissue under the evaluated conditions. In contrast, lead (Pb) exhibited considerable inter-individual variability, with sporadically elevated concentrations most likely associated with secondary contamination from bullet fragments. Although the analysed muscle samples generally demonstrated favourable nutritional characteristics, the occurrence of elevated Pb concentrations highlight the importance of continued monitoring and appropriate carcass handling practices. Because the present study was based on a limited number of opportunistically obtained individuals sampled during the autumn pre-hibernation period, further investigations involving larger datasets and seasonal comparisons are warranted.

## Data Availability

No datasets were generated or analysed during the current study.
